# Genotypic Analysis of *Klebsiella pneumoniae Isolates* in a Beijing Hospital Reveals High Genetic Diversity and Clonal Population Structure of Drug-Resistant Isolates

**DOI:** 10.1371/journal.pone.0057091

**Published:** 2013-02-21

**Authors:** Qi Wang, Bin Li, Alan K. L. Tsang, Yong Yi, Patrick C. Y. Woo, Cui Hua Liu

**Affiliations:** 1 CAS Key Laboratory of Pathogenic Microbiology and Immunology, Institute of Microbiology, Chinese Academy of Sciences, Beijing, China; 2 State Key Laboratory of Emerging Infectious Diseases, Department of Microbiology, The University of Hong Kong, Hong Kong Special Administrative Region, China; 3 The 306 Hospital, Beijing, China; University of Padova, Medical School, Italy

## Abstract

**Background:**

The genetic diversity and the clinical relevance of the drug-resistant *Klebsiella pneumoniae* isolates from hospital settings are largely unknown. We thus conducted this prospective study to analyze the molecular epidemiology of *K. pneumoniae* isolates from patients being treated in the 306 Hospital in Beijing, China for the period of November 1, 2010–October 31, 2011.

**Methodology/Principal Findings:**

Antibiotic susceptibility testing, PCR amplification and sequencing of the drug resistance-associated genes, and multilocus sequence typing (MLST) were conducted. A total of 163 isolates were analyzed. The percentage of MDR, XDR and PDR isolates were 63.8% (104), 20.9 (34), and 1.8% (3), respectively. MLST results showed that 60 sequence types (STs) were identified, which were further separated by eBURST into 13 clonal complexes and 18 singletons. The most dominant ST was ST15 (10.4%). Seven new alleles and 24 new STs were first identified in this study. Multiple logistic regression analysis revealed that certain clinical characteristics were associated with those prevalent STs such as: from ICU, from medical ward, from community acquired infection, from patients without heart disease, from patients with treatment success, susceptible to extended spectrum cephalosporin, susceptible to cephamycins, susceptible to fluoroquinolones, and with MDR.

**Conclusions/Significance:**

Our data indicate that certain drug-resistant *K. pneumoniae* clones are highly prevalent and are associated with certain clinical characteristics in hospital settings. Our study provides evidence demonstrating that intensive nosocomial infection control measures are urgently needed.

## Introduction

*Klebsiella pneumoniae* is an important bacterial pathogen associated with community acquired (CA) and hospital acquired (HA) infections and has the potential to cause severe morbidity and mortality, particularly in immunocompromised patients [1]–[3]. Infections caused by drug-resistant *K. pneumoniae* isolates, especially those produce extended-spectrum beta-lactamases (ESBLs) and which are multidrug-resistant (MDR), extensively drug-resistant (XDR) or pandrug-resistant (PDR), are more difficult and expensive to treat with worse treatment outcome [4]–[8]. More recently, carbapenem-resistant *K. pneumoniae* have been reported worldwide as a consequence of acquisition of carbapenemase genes, and a large variety of carbapenemases have been identified in *K. pneumoniae* [9]–[14].

Rapid and discriminative genotyping methods are useful for determining the clonality of the isolates in nosocomial or household outbreaks [15], [16]. Multilocus sequencing typing (MLST) is a nucleotide sequence-based approach for characterizing bacterial isolates (http://www.mlst.net/), with the advantage over traditional pulsed-field gel electrophoresis (PFGE) of ease of manipulation and convenient comparison [17], [18]. In our previous study, we observed alarmingly high rates of MDR, XDR and PDR strains among *K. pneumoniae* isolates from a tertiary care hospital in Beijing, China [19]. In addition, data from that study indicate that many of the drug resistance genes were transmissible [19]. Since the genetic diversity, transmission patterns and the clinical relevance of the drug-resistant *K. pneumoniae* isolates from hospital settings are largely unknown, we thus further conducted this MLST genotyping analysis for *K. pneumoniae* isolates from the 306 Hospital, a tertiary care hospital in Beijing, China for the period of November 1, 2010–October 31, 2011 with an aim to assess the molecular epidemiology as well as clinical characteristics associated with prevalent *K. pneumonia*e clones.

## Methods

### Ethics statement

All of the investigation protocols in this study were approved by the institutional ethics committee of the 306 Hospital, Beijing, China. Written informed consent for *K. pneumoniae* isolates to be collected as well as for their information to be stored in the hospital database for research purposes was provided by participants. Written informed consent was obtained from the next of kin, caretakers, or guardians on the behalf of the minors/children participants involved in this study. Permission for using the information in the medical records of the patients for research purposes was obtained from the 306 Hospital. The Institute ethics committee of the 306 Hospital reviewed that relevant ethical issues in this study were considered.

### Study population, bacterial isolate identification, and drug susceptibility testing

The 306 Hospital in Beijing, China is a tertiary care hospital, with 1,100 beds and approximately 25,000 hospital admissions per year. Consecutive non-repetitive *K. pneumoniae* isolates were collected from patients being treated in the 306 Hospital for the period of November 1, 2010–October 31, 2011. Isolates with ambiguous sequence data for one or more alleles were excluded from the analysis. All isolates were cultured in Luria-Bertani (LB) medium. A total of 175 isolates were confirmed as *K. pneumoniae* by 16S rDNA sequencing. Drug susceptibility testing (DST) for the *K. pneumoniae* isolates was performed using the bioMérieux VITEK2 system following manufacturer's instructions. The following 18 drugs were tested: ampicillin (AMP), piperacillin/tazobactam (TZP), ampicillin/sulbactam (SAM), cefazolin (CFZ), ceftriaxone (CRO), ceftazidime (CAZ), cefepime (FEP), cefotetan (CTT), ertapenem (ETP), imipenem (IM), aztreonam (ATM), ciprofloxacin (CIP), levofloxacin (LVX), gentamicin (GM), tobramycin (TOB), amikacin (AMK), trimethoprim-sulfamethoxazole (SXT), and nitrofurantoin (FD). The ESBLs were detected by the bioMérieux VITEK-2 AST-GN13 test. In some cases, the ESBL positivity was further confirmed by the double disk diffusion method according to standard protocols by the Clinical Laboratory Standard Institute (CLSI) [20]. *Escherichia coli* strains ATCC 25922 and ATCC 35218, *Klebsiella pneumoniae* strain ATCC 700603 and *Pseudomonas aeruginosa* strain ATCC 27853 were used as quality control strains for the DST. Clinical records of patients from whom the *K. pneumoniae* isolates were obtained were reviewed retrospectively.

### PCR amplification and sequencing for drug resistance genes

Drug resistance-associated genes were detected by PCR and sequencing using 37 pairs of primers as described previously [19]. The drug resistance-associated genes examined include: *bla*_CTX-M-1_, *bla*_CTX-M-2_, *bla*_CTX-M-3_, *bla*_CTX-M-8_, *bla*_CTX-M-9_, *bla*_CTX-M-10_, *bla*_CTX-M-14_, *bla*_CTX-M-25_, *bla*_SHV-group_, *bla*_TEM_, *bla*_KPC_, *bla*_NDM_, *bla*_IMP_, *bla*_VIM_, *bla*_OXA-48_, *bla*_CMY_, *bla*_DHA_, *bla*_FOX_, *dhfr*, *qnrA*, *qnrB*, *qnrC*, *qnrD*, *qnrS*, *aac(6′)-Ib-cr*, *qepA*, *gyrA*, *parC*, *aacA4*, *aacC1*, *aacC2*, *aadA1*, *aadB*, *aphA6*, *armA*, *rmtB*, and *Integron I*. DNA sequences were annotated using the BLAST program at http://www.ncbi.nlm.nih.gov.

### Genotyping of *K. pneumoniae* isolates by MLST analysis

Genotyping was determined by MLST analysis. MLST with seven genes (*gapA*, *infB*, *mdh*, *pgi*, *phoE*, *rpoB* and *tonB*) was performed on isolates according to the protocol described on the *K. pneumoniae* MLST website (www.pasteur.fr/mlst) [18]. Alleles and sequence types (STs) were assigned by using the MLST database (www.pasteur.fr/mlst/Kpneumoniae.html). Alleles and STs that had not been previously described were submitted to the curator of the database and were assigned new designations.

### Assignment to clonal complexes

The program eBURST v 3.0 was used to identify the different clonal complexes [21]. Clonal complexes were defined as groups of two or more independent isolates that shared identical alleles at six or more loci; each complex was named after the putative founder ST. Data from additional 1,380 isolates of *K. pneumonia*e were obtained from the MLST isolate database deposited at the Pasteur Institute (http://www.pasteur.fr/cgi-bin/genopole/PF8/mlstdbnet.pl?file=klebs_isolates.xml) [18].

### Sequence analysis

The proportions of nucleotide alterations that led to a change in the amino acid sequence (non-synonymous substitution, *dn*) and the proportions of nucleotide alterations that did not lead to a change in the amino acid sequence (synonymous substitution, *ds*) were calculated with START2 [22]. Phylogenetic analysis was performed using ClonalFrame algorithm with the software package ClonalFrame version 1.1 [23], using 50,000 burn-in cycles and 100,000 further iterations. Maximum likelihood tree was constructed using PhyML 3.0 under a GTR+I+G model based on the alignment of concatenated sequence from the seven MLST gene loci with 1000 bootstrap replicates [24].

### Measurement of clonality

The standardized index of association (*I_A_^S^*) for the seven loci was calculated using START2 software and 1,000 iterations [22]. *I_A_^S^* was also estimated using 764 unique STs including the new STs in this study and existing STs in the entire MLST isolate database as a whole.

### Definitions

Every patient was counted only once during his hospital stay regardless of the number of positive cultures. Each case was differentiated between CA infection and HA infection based on a temporal definition. A CA case was defined as a case with known carriage of *K. pneumoniae* on admission or with the first culture positive for these bacteria within 48 h of admission [6], [25]. A HA case was defined by the first culture positive obtained more than 48 h after admission. MDR was defined as acquired non-susceptibility to at least one agent in three or more antimicrobial categories, XDR was defined as non-susceptibility to at least one agent in all but two or fewer antimicrobial categories (i.e. bacterial isolates remain susceptible to only one or two categories) and PDR was defined as non-susceptibility to all agents in all antimicrobial categories [7].

### Statistical analyses

Data were entered and analysed using the statistical package SPSS for windows (version 15). Two people were independently cross-checked each entry to ensure the quality of data entered into the computer. For categorical data, different groups were compared using the Chi-square test. Univariate and multivariate analyses were used to determine the factors associated with prevalent *K. pnuemoniae* clones. The factors examined were shown in Table S5. All Potentially associated factors were included in a logistic regression model for multivariate analysis, and they were eliminated using a backward stepwise selection method using a *P* value threshold of 0.1 for the variables to be remained in the model. Mantel-Haenszel odds ratios (ORs), 95% confidence intervals (CIs) and corresponding *P* values were reported. *P* value of <0.05 was considered to be statistically significant.

## Results

### Demographic and clinical characteristics of the patients

**Figure 1:**
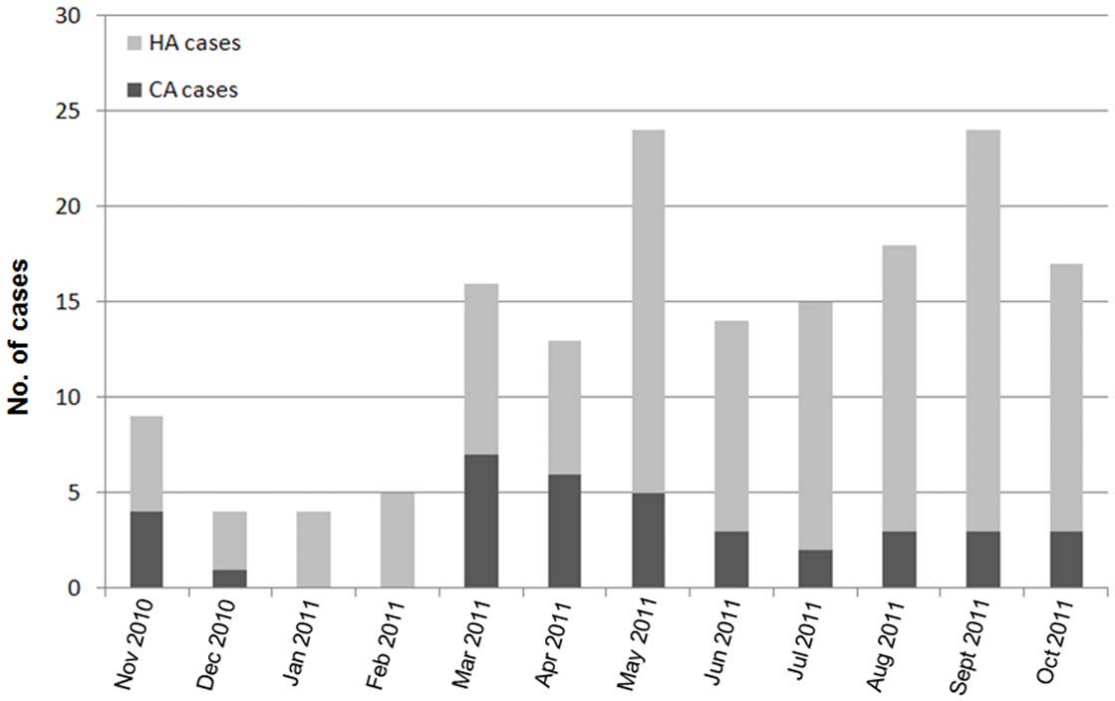
Epidemiology of hospitalized patients that were colonized or infected with *K. pneumoniae* isolates in the 306 Hospital during November 1, 2010–October 31, 2011. Note: HA: hospital acquired; CA: community acquired.

From November 1, 2010 to October 31, 2011, a total of 175 non-repetitive hospitalized patients who had *K. pneumoniae* isolates available were subjected to DST at the 306 Hospital. Twelve (6.9%) patients were excluded from the study as a result of low-quality sequencing results for one or more of the 7 house-keeping genes of their isolates. Among the remaining 163 patients, 126 (77.3%) were HA cases and 37 (22.7%) were CA cases. Eighty (49.1%) isolates were ESBL positive and 83 (50.9%) isolates were ESBL negative. The proportion of the male and female patients were 73.6% (120/163) and 26.4% (43/163), respectively. Fifty-four (33.1%) of the patients were Beijing residents and the rest were from other provinces of China (non-Beijing residents). The median (±SD) age of the patients was 74.50±19.16 years (range 1–98 years). The majority of the patients were from medical ward (75/163, 46.0%) and intensive care unit (ICU) (49/163, 30.1%). The main source of the specimens was sputum (121/163, 74.2%). The proportion of MDR, XDR, PDR, and other types of *K. pneumoniae* isolates were 63.8% (104/163), 20.9% (34/163), 1.8% (3/163) and 13.5% (22/163) respectively. Notably, the rates of resistance to most drugs were much higher among ESBL positive isolates than ESBL negative isolates. The epidemiology of CA and HA cases based on the admission date is shown in Fig. 1. More detailed information on relevant demographic and clinical characteristics of the study population is summarized in Table 1.

**Table 1:** Demographic and clinical characteristics of the patients.

Characteristics	Total n = 163 (%)	ESBL positive cases n = 80 (%)	ESBL negative cases n = 83 (%)	*P* value
Gender				0.166
Male	120 (73.6)	55 (68.8)	65 (78.3)	
Female	43 (26.4)	25 (31.3)	18 (21.7)	
Age group, years				
<15	6 (3.7)	3 (3.8)	3 (3.6)	0.963
15–60	31 (19.0)	14 (17.5)	17 (20.5)	0.628
>60	126 (77.3)	63 (78.8)	63 (75.9)	0.665
Residence situation				0.406
Beijing resident	54 (33.1)	29 (36.3)	25 (30.1)	
Non-Beijing resident	109 (66.9)	51 (63.8)	58 (69.9)	
Hospital location				
Emergency room	8 (4.9)	3 (3.8)	5 (6.0)	0.502
Intensive care unit	49 (30.1)	27 (33.8)	22 (26.5)	0.313
Medical ward	75 (46.0)	37 (46.3)	38 (45.8)	0.952
Surgical ward	31 (19.0)	13 (16.3)	18 (21.7)	0.377
Sources of specimens				
Sputum	121 (74.2)	60 (75.0)	61 (73.5)	0.826
Urine	12 (7.4)	9 (11.3)	3 (3.6)	0.062
Throat or nose swabs	13 (8.0)	3 (3.8)	10 (12.0)	0.051
Blood	8 (4.9)	5 (6.3)	3 (3.6)	0.436
Others	9 (5.5)	3 (3.8)	6 (7.2)	0.331
Underlying diseases				
Pneumonia	14 (8.6)	6 (7.5)	8 (9.6)	0.610
Diabetes mellitus	40 (24.5)	19 (23.8)	21 (25.3)	0.786
Chronic bronchitis	11 (6.7)	6 (7.5)	5 (6.0)	0.721
Chronic obstructive pulmonary disease	14 (8.6)	7 (8.8)	7 (8.4)	0.959
Abnormal liver function	36 (22.1)	13 (16.3)	23 (27.7)	0.070
Renal dysfunction	35 (21.5)	21 (26.3)	14 (16.9)	0.152
Hypertension	84 (51.5)	39 (48.8)	45 (54.2)	0.427
Heart disease	55 (33.7)	30 (37.5)	25 (30.1)	0.337
Cerebral infarction	41 (25.2)	22 (27.5)	19 (22.9)	0.520
Pulmonary infection	81 (49.7)	42 (52.5)	39 (47.0)	0.513
Urinary tract infection	11 (6.7)	4 (5.0)	7 (8.4)	0.383
Infection acquired model				0.665
Community acquired	37 (22.7)	17 (21.3)	20 (24.1)	
Hospital acquired	126 (77.3)	63 (78.8)	63 (75.9)	
Drug resistance profiles				
Penicillins	162 (99.4)	80 (100.0)	82 (98.8)	0.325
1st and 2nd generation cephalosporins	86 (52.8)	78 (97.5)	8 (9.6)	<0.001
3rd and 4th generation cephalosporins	86 (52.8)	77 (96.3)	9 (10.8)	<0.001
Cephamycins	108 (66.3)	37 (46.3)	71 (85.5)	<0.001
Carbapenems	12 (7.4)	8 (10.0)	4 (4.8)	0.205
Monobactams	83 (50.9)	77 (96.3)	6 (7.2)	<0.001
Fluoroquinolones	72 (44.2)	53 (66.3)	19 (22.9)	<0.001
Aminoglycosides	90 (55.2)	71 (88.8)	19 (22.9)	<0.001
Folate pathway inhibitors	94 (57.7)	76 (95.0)	18 (21.7)	<0.001
Nitrofurantoin	145 (89.0)	77 (96.3)	68 (81.9)	0.004
Drug resistance types				
MDR-KP	104 (63.8)	49 (61.3)	55 (66.3)	0.505
XDR-KP	34 (20.9)	22 (27.5)	12 (14.5)	0.040
PDR-KP	3 (1.8)	3 (3.8)	0	0.075
Other types of KP	22 (13.5)	6 (7.5)	16 (19.3)	0.028
Treatment outcome				0.751
Treatment success	138 (84.7)	67 (83.8)	71 (85.5)	
Died	25 (15.3)	13 (16.3)	12 (14.5)	

### MLST analysis of the *K. pneumoniae* isolates and identification of clonal complexes

**Figure 2:**
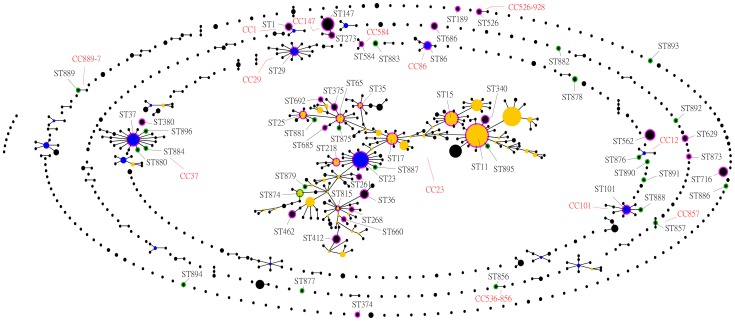
Comparative eBURST analysis showing the clonal assignment of the STs present in this study compared to that of the STs in the entire *K. pneumonia* MLST isolate database. Only STs in this study are given, and lines connect single locus variants. The names of the CCs are based on the ST assigned as the founder genotype of the complex shown in blue. Subgroup founders are shown in yellow. The relative size of the circles indicates their prevalence. New STs identified in this study are highlighted by a green halo; STs present in the database and this study are highlighted by a pink halo.

**Figure 3:**
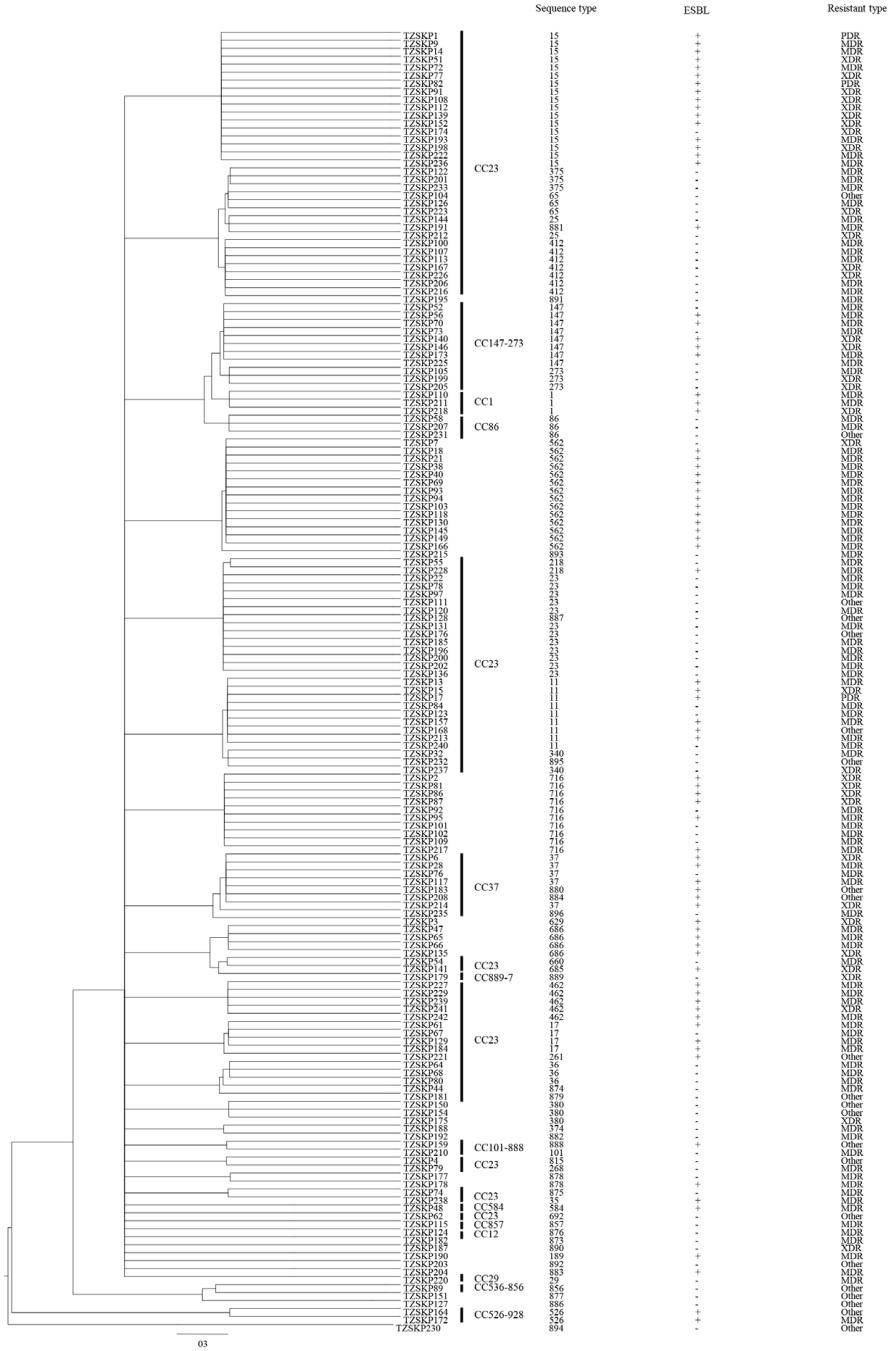
50% majority-rule consensus phylogenetic tree derived from *ClonalFrame* for the 7 housekeeping loci in *K. pneumoniae*, displaying the clonal relationship between the STs and drug resistance of the *K. pneumoniae* population.

MLST was conducted to determine the extent of genotypic diversity among the *K. pneumoniae* isolates. Sixty different STs were identified by MLST. Thirty-seven of the STs contained single isolates, while 23 STs included between 2 and 17 isolates. 24 (40%) of the 60 STs had not been previously identified, 16 of these differed from recognized STs at only a single locus, 5 of these (ST877, ST882, ST883, ST890 and ST891) differed from recognized STs at two loci, and 3 of these (ST886, ST893 and ST894) differed from recognized STs at three loci (Table S1). The 60 STs generated in this data set were separated by eBURST into 13 clonal complexes and 18 singletons with the default stringent definition of the groups by sharing alleles at 6 of 7 loci (Fig. 2 and 3, Table 2, and Table S1). The most dominant ST was ST15 (10.4%, 17/163), followed by ST562 (8.6%, 14/163), ST23 (7.4%, 12/163), ST716 (6.1%, 10/163), ST11 (5.5%, 9/163), ST147 (4.9%, 8/163). Those 6 STs accounted for 42.9% (70/163) of the total isolates, and those 70 isolates were thus designated prevalent clones in this study. Two of the three PDR isolates in this study belonged to ST15.

**Table 2:** Distribution of sequence types in *K. pneumoniae* clonal complexes.

Clonal complex	Sequence type	Allelic profile	No. of isolates		No. of isolates		
			ESBL+		ESBL -	HA	CA
			*bla*_CTX-M_	*bla*_TEM_/*bla*_SHV_			
CC23	23	2-1-1-1-9-4-12	0	0	12	7	5
	11	3-3-1-1-1-1-4	5	4	3	5	4
	15	1-1-1-1-1-1-1	14	11	1	10	7
	17	2-1-1-1-4-4-4	3	2	1	3	1
	65	2-1-2-1-10-4-13	0	0	3	3	0
	340	3-3-1-1-1-1-18	0	0	2	2	0
	895	3-3-1-1-1-1-42	0	0	1	1	0
	815	2-1-2-1-7-1-12	0	0	1	1	0
	35	2-1-2-1-10-1-19	1	1	0	1	0
	268	2-1-2-1-7-1-81	0	0	1	0	1
	875	2-1-2-1-10-4-19	0	0	1	0	1
	25	2-1-1-1-10-4-13	0	0	2	1	1
	36	2-1-2-1-7-1-7	0	0	3	3	0
	218	2-3-1-1-9-4-12	1	0	1	1	1
	375	43-1-2-1-10-4-13	0	0	3	3	0
	412	2-1-2-1-9-1-112	0	0	7	6	1
	660	2-1-2-1-4-1-25	0	0	1	0	1
	685	2-1-2-1-3-4-25	1	1	0	1	0
	874	4-1-1-1-7-1-22	1	0	1	1	0
	887	2-1-1-1-21-4-12	0	0	1	1	0
	692	2-1-2-1-1-4-42	0	0	1	0	1
	879	2-1-1-1-145-1-9	0	0	1	1	0
	261	2-1-1-1-4-27-12	0	0	0	1	0
	462	2-1-1-6-1-4-12	4	2	0	3	2
	881	2-68-1-1-10-4-13	1	1	0	1	0
CC37	37	2-9-2-1-13-1-16	3	4	1	3	2
	896	2-9-2-1-13-1-38	0	0	1	1	0
	884	77-9-2-1-13-1-16	1	1	0	1	0
	880	2-9-2-1-1-1-16	1	1	0	1	0
CC29	29	2-3-2-2-6-4-4	0	0	1	1	0
CC101	101	2-6-1-5-4-1-6	0	0	1	1	0
	888	2-6-1-5-4-1-4	1	1	0	1	0
CC86	86	9-4-2-1-1-1-27	0	0	3	3	0
CC1	1	4-4-1-1-7-4-10	3	2	0	2	1
CC12	876	6-3-1-1-12-1-110	0	0	1	1	0
CC147	147	3-4-6-1-7-4-38	5	3	3	5	3
	273	3-4-6-1-7-4-4	0	0	3	2	1
CC857	857	2-35-2-35-56-24-19	0	0	1	1	0
CC584	584	4-1-2-1-1-7-4	1	1	0	1	0
CC526-928	526	38-19-53-58-73-21-130	2	2	0	2	0
CC536-856	856	16-18-21-27-39-22-105	0	0	1	1	0
CC889-7	889	3-1-1-4-3-1-19	0	0	1	0	1
Singleton	894	18-15-18-61-93-37-99	0	0	1	1	0
Singleton	893	25-1-101-1-10-1-100	0	0	1	1	0
Singleton	892	2-1-65-2-5-4-36	0	0	1	1	0
Singleton	891	3-1-2-1-9-1-184	0	0	1	1	0
Singleton	890	2-1-1-8-10-4-61	0	0	1	1	0
Singleton	886	16-1-21-27-29-17-183	0	0	1	1	0
Singleton	883	2-5-1-1-9-4-9	1	1	0	0	1
Singleton	882	2-1-1-37-1-27-19	0	0	1	1	0
Singleton	878	2-5-1-1-144-1-4	1	1	1	2	0
Singleton	877	59-24-21-78-54-22-67	0	0	1	0	1
Singleton	189	2-3-41-1-17-4-46	1	0	0	1	0
Singleton	374	2-3-58-37-10-27-9	0	0	1	1	0
Singleton	380	2-1-1-1-1-4-19	0	0	3	3	0
Singleton	562	2-5-1-1-10-1-139	9	8	1	13	1
Singleton	629	2-3-87-1-12-1-26	1	1	0	1	0
Singleton	686	4-1-1-3-3-5-54	4	3	0	4	0
Singleton	716	71-1-1-2-16-4-164	5	5	4	10	0
Singleton	873	14-1-2-1-7-4-182	0	0	1	1	0

### Measurement of clonality and selection pressure

Analysis of the data set of 163 isolates from patients yielded an *I_A_^S^* value of 0.1251. This was decreased to 0.0841 when only one representative of each sequence type was included. Significant linkage disequilibrium was detected in both analyses. It remained significant when only one representative isolate for each ST in the entire isolate database were considered (*I_A_^S^* = 0.1133), thus the observed linkage disequilibrium is not due to sampling bias. The values of *I_A_^S^* in these analyses were low suggesting the weakly clonal population. The *dn*/*ds* ratios for all loci were significantly less than 1 (Table S2), indicating there was no strong positive selective pressure on the genes.

### Phylogenetic analysis

**Figure 4:**
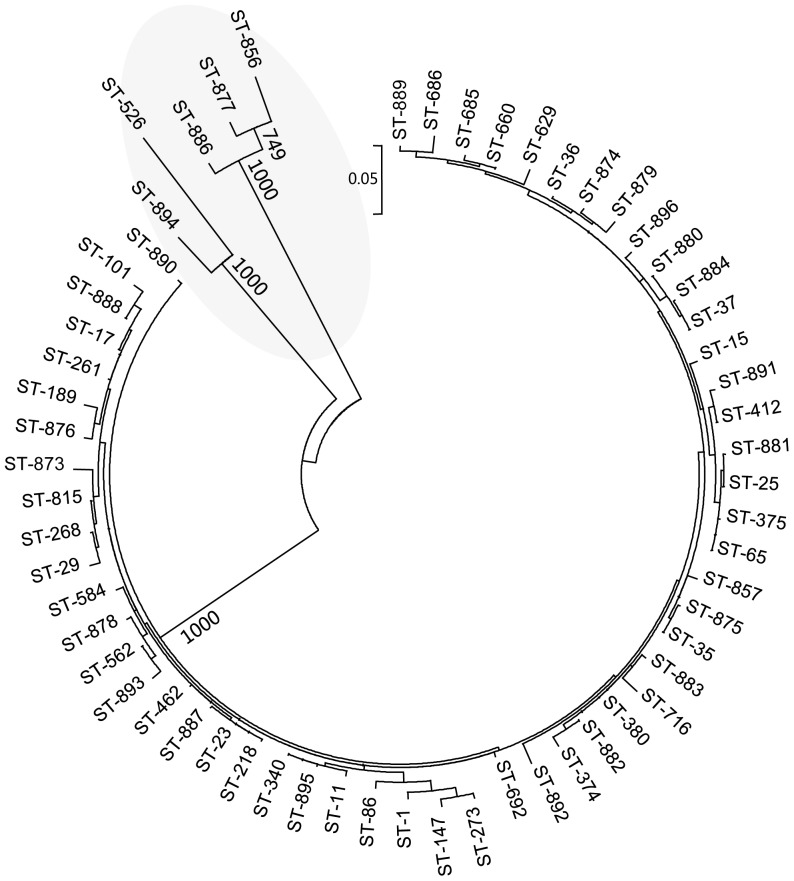
Phylogenetic tree of *K. pneumoniae* isolates with one representative in each ST as derived from concatenated sequences of the 7 gene loci used in MLST. The tree was constructed using the maximum likelihood method. The phylogenetically distinct cluster is shaded.

The maximum likelihood tree show a phylogenetically distinct cluster of related STs (ST877, ST886, ST894, ST856 and ST526) is formed with strong bootstrap support. Four of them (ST877, ST886, ST894 and ST856) are new STs in this study, and 2 of these (ST886 and ST894) differed from recognized STs at three loci (Fig. 4). The drug resistance profiles and epidemiological information of those clones belonging to phylogenetically distinct cluster of related STs are shown in Table S3.

### Drug resistance profiles of *K. pneumoniae* isolates grouped by CCs and STs

The detailed information on drug resistance profiles of *K. pneumoniae* isolates grouped by CCs is shown in Table 3. All CCs showed high proportion of resistance to penicillin. We did not observe significant differences between ESBL blaCTX-M and blaTEM/SHV groups (Table 2 and Table 3). We also compared the drug resistance profiles, the corresponding drug resistance-associated genes, as well as the clinical characteristics of the prevalent *K. pneumoniae* clones (Table S4). We observed that some isolates with the same STs were from patients who were hospitalized in the same period and who shared the same wards, especially in the first ward of the department of respiration, neuro-intensive care unit (NICU), and ICU. In addition, we detected a large variety of ESBL genes (such as *bla*_SHV_ and *bla*_CTX-M_, and *bla*_TEM_), as well as genes associated with resistance to fluoroquinolones (such as *qnrA*, *qnrB*, *qnrC*, *qnrD*, *qnrS*, *aac(6′)-Ib-cr*, and *qepA*) and aminoglycosides (such as *aacC2*, *addA1*, and *aacA4*) in the prevalent clones. We detected the *bla*_OXA-48_ gene in one of the PDR *K. pneumoniae* isolate (TZSKP-82).

**Table 3:** Drug resistance profiles of *K. pneumoniae* isolates grouped by clonal complex.

Clonal complex (No. of isolates)	Drug resistance types, No. (%) of isolates			Drug resistance, No. (%) of isolates										
	MDR	XDR	PDR	Penicillin	Non-extended spectrum cephalosporins	Extended spectrum cephalosporins(*bla*_CTX-M_)	Extended spectrum cephalosporins(*bla*_TEM_/*bla*_SHV_)	Cephamycins	Carbapenems	Monobactams	Fluoroquinolones	Aminoglycosides	Folate pathway inhibitors	Nitrofurantoin
CC1 (3)	2(66.7)	1(33.3)	0	3 (100.0)	3 (100.0)	3(100.0)	2 (66.7)	3 (100.0)	1(33.3)	3(100.0)	3(100.0)	2 (66.7)	2 (66.7)	3(100.0)
CC12 (1)	1(100.0)	0	0	1(100.0)	0	0	0	1(100.0)	0	0	0	0	0	1(100.0)
CC23 (82)	52(63.4)	17(20.7)	3(3.7)	82 (100.0)	39 (47.6)	33 (40.2)	26 (31.7)	61(74.4)	6(7.3)	39(47.6)	34(41.5)	41(50.0)	42(51.2)	73(89.0)
CC29 (1)	1(100.0)	0	0	1(100.0)	0	0	0	0	0	0	0	0	0	1(100.0)
CC37 (8)	4 (50.0)	2 (25.0)	0	8(100.0)	6 (75.0)	5 (62.5)	6 (75.0)	4(50.0)	0	6(75.0)	3(37.5)	5(62.5)	4(50.0)	7(87.5)
CC86 (3)	2(66.7)	0	0	3(100.0)	0	0	0	2(66.7)	0	0	0	0	0	1(33.3)
CC101 (2)	1(50.0)	0	0	2(100.0)	1(50.0)	1(50.0)	1(50.0)	1(50.0)	0	1(50.0)	0	1(50.0)	1(50.0)	2(100.0)
CC147 (11)	7 (63.6)	4 (36.4)	0	11(100.0)	8 (72.7)	4 (36.4)	5 (45.5)	7 (63.6)	1(9.1)	5 (45.5)	11(100.0)	10(90.9)	11(100.0)	11(100.0)
CC526-928 (2)	1 (50.0)	0	0	2(100.0)	2(100.0)	2(100.0)	2(100.0)	0	0	2(100.0)	2(100.0)	2(100.0)	2(100.0)	2(100.0)
CC536-856 (1)	0	0	0	1(100.0)	0	0	0	0	0	0	0	0	0	0
CC584 (1)	1(100.0)	0	0	1(100.0)	1(100.0)	1(100.0)	1(100.0)	0	0	1(100.0)	0	1(100.0)	1(100.0)	1(100.0)
CC857 (1)	1(100.0)	0	0	1(100.0)	0	0	0	1(100.0)	0	0	0	0	0	1(100.0)
CC889-7 (1)	0	1(100.0)	0	1(100.0)	0	0	0	1(100.0)	0	0	0	0	0	1(100.0)
Singletons(46)	31(67.4)	9(19.6)	0	45(97.8)	26 (56.5)	21(45.7)	19(41.3)	27(58.7)	3(6.5)	26(56.5)	19(41.3)	28(60.9)	31(67.4)	41(89.1)
Total(163)	104(63.8)	34(20.9)	3(1.8)	162(99.4)	86(52.8)	70(42.9)	62(38.0)	108(66.3)	11(6.7)	83(50.9)	72(44.2)	90(55.2)	94(57.7)	145(89.0)

### Factors associated with prevalent *K. pneumoniae* clones

Factors associated with prevalent STs compared with non-prevalent STs upon univariate and multivariate analysis are shown in Table S5 and Table 4, respectively. Multiple logistic regression analysis revealed that isolates from ICU (OR, 13.802), from medical ward (OR, 5.154), from community acquired infection (OR, 3.106), from patients without heart disease (OR, 3.446), from patients with treatment success (OR, 6.691), susceptible to extended spectrum cephalosporins (OR, 8.633), susceptible to cephamycins (OR, 3.430), susceptible to fluoroquinolones (OR, 6.247), with MDR (OR, 3.111) were significantly associated with the prevalent STs.

**Table 4:** Multivariate logistic regression analysis for factors independently associated with prevalent *K. pneumoniae* clones^a^.

Variables^b^	Univariate analysis		Multivariate analysis Univariate analysis	
	Odds Ratio (95% CI)	*P* value	Odds Ratio (95% CI)	*P* value
From intensive care unit	1.597 (0.814–3.135)	0.174	13.802 (3.180–59.891)	<0.001
From medical ward	1.198 (0.643–2.232)	0.570	5.154 (1.452–18.296)	0.011
Community acquired infection	1.788 (0.854–3.742)	0.123	3.106 (1.005–9.602)	0.049
With diabetes mellitus	1.244 (0.603–2.565)	0.554	2.776 (0.981–7.854)	0.054
With abnormal liver function	1.630 (0.770–3.452)	0.202	2.551 (0.924–7.042)	0.071
With cerebral infarction	1.529 (0.746–3.136)	0.246	2.528 (0.926–6.900)	0.070
Without heart disease	1.250 (0.640–2.440)	0.513	3.446 (1.298–9.150)	0.013
Treatment success	1.408 (0.582–3.406)	0.447	6.691 (1.884–23.770)	0.003
Susceptible to 3rd and 4th generation cephalosporins	4.448 (2.268–8.722)	<0.001	8.633 (2.515–29.640)	0.001
Susceptible to cephamycins	1.072 (0.556–2.068)	0.836	3.430 (1.078–10.921)	0.037
Susceptible to fluoroquinolones	5.558 (2.823–10.943)	<0.001	6.247 (2.117–18.439)	0.001
MDR	1.291 (0.674–2.473)	0.442	3.111 (1.113–8.693)	0.030

a Prevalent clones include a total of 70 isolates with the following STs: ST15 (17), ST562 (14), ST23 (12), ST716 (10), ST11 (9), ST147 (8).

b All variables included in the univariate analysis (shown in Table S5) were included in the logistic regression model for multivariate analysis, and they were eliminated using a backward stepwise selection method using a *P* value threshold of 0.1 for the variables to remain in the model. *P* values<0.05 were considered to be statistically significant.

## Discussion

The present study describes the genetic diversity of drug-resistant *K. pneumoniae* isolates in a tertiary hospital in Beijing. Twenty-four new STs were detected, demonstrating that the MLST database is still novel and continuously growing. The isolates originated from both CA and HA infections. It is noteworthy that in contrast to the observations from a study from Germany [1], the levels of resistance were equally high among both HA and CA *K. pneumoniae* isolates in our study, indicating a very large reservoir of resistance in the community around Beijing. We further analyzed the drug resistance profiles, the corresponding drug resistance-associated genes, as well as the clinical characteristics of those isolates with the same STs. We observed that the rates of resistance to most drugs were much higher among ESBL positive isolates than ESBL negative isolates. But we did not observe significant differences between ESBL blaCTX-M and blaTEM/blaSHV groups in their distribution of STs or CCs. We noticed that some prevalent isolates with the same STs were from patients who were hospitalized in the same period and who shared the same wards, especially in the first ward of the department of respiration, NICU, and ICU, this observation suggests that those clonal isolates were transmitted in the hospital, causing infections among immunocompromised patients in those wards. In addition, many patients with this ST shared the same wards such as the first ward of the department of respiration and cardiac care unit, suggesting there is currently an ongoing transmission of isolates of this ST in those wards of the hospital.

Notably, among the three isolates of PDR *K. pneumoniae*, two isolates belonged to ST15, which is the most prevalent ST in this study. The clinical and molecular epidemiological data suggest that the majority of those ST15 isolates were from patients with severe underlying diseases such as pulmonary infection, renal dysfunction, heart failure, and chronic obstructive pulmonary disease, etc. A study from Spain reported that VIM-1 producing *K. pneumoniae* ST15 clone has a high capacity to spread among ICU patients with severe underlying conditions [2]. ST15 is also widespread in other countries such as Denmark, Hungary, Korea, Malaysia, Singapore and Taiwan [26]–[28]. Interestingly, one of the PDR *K. pneumoniae* isolate (TZSKP-82) possesses the *bla*_OXA-48_ gene. OXA-48 carbapenemases were first isolated from *K. pneumoniae* in Turkey in 2008 [10], [29]. To the best of our knowledge, this is the first documented case of OXA-48-producing *K. pneumoniae* in China.

ST23 was another prevalent ST in this study. ST23 was the primary founder of CC23, and isolates sharing this ST were found in other countries. Previous studies showed that ST23 isolates were closely related to liver abscess [30], [31]. However, the ST23 isolates in this study were diagnosed with different kinds of illnesses including cerebral infarction, renal dysfunction, and liver abscess, etc.

Another frequently identified ST is ST11, which is a single locus variant of ST258. ST258 is a well known lineage of *K. pneumoniae* which plays an important role in the global spread of carbapenemases. ST258 was not found in our study. There are only a few nucleotide differences between ST11 and ST258 in their *tonB* alleles. ST258 was proposed to be probably arisen from ST11 by acquisition of the *tonB*-79 allele, followed by acquisition of carbapenem-resistance genes on mobile elements [32]. A recent study was conducted to analyze carbapenem-resistant *K. pneumoniae* isolates from 13 hospitals in nine cities covering five provinces in China, and they found that ST11 was the most dominant clone among the 95 carbapenem-resistant *K. pneumoniae* isolates in China [33]. Although ST11 is not the most dominant one in our study, it is among one of the prevalent clones and all those ST11 isolates harbored ESBL genes. In addition, the genetic relatedness of ST11 with ST258 is of great concern.

From the maxiumum likelihood tree, a phylogenetically distinct cluster of related STs (ST877, ST886, ST894, ST856 and ST526) is formed. Four of them (ST877, ST886, ST894 and ST856) are new STs in this study, and 2 of these (ST886 and ST894) differed from recognized STs at three loci. These results suggest a recent clone is emerging locally. Further identification of the drug resistance profiles and epidemiological information of the 4 isolates with new STs imply that they are relatively susceptible (with resistance to only a few drugs and no resistance genes detected).

One of the challenges for infection control is to discern the prevalent clones as well as their clinical relevance, especially the treatment outcome, of those isolates, so as to provide information for better management measures. We thus further examined the association between certain prevalent *K. pneumoniae* isolates (based on the frequency of the STs) and the demographic and clinical features as well as mortality of the patients from whom the isolates were obtained. We noticed that some results (For example, the results for the association with the intensive care unit) from univariant and multivariant analysis differ greatly. We think that the results from the multivariant analysis should be more reliable and those variables with significant *P* values in the multivariant analysis were identified to be independently associated with prevalent *K. pneumoniae* clones after excluding some less significant variables and taking into consideration of the confounding factors during the multivariate analysis. Data from multiple logistic regression analysis revealed that isolates from ICU, from medical ward, from community acquired infection, and with MDR were significantly associated with those prevalent clones. In addition, we noticed that those prevalent clones were more frequently associated with patients without heart disease, who were susceptible to extended spectrum cephalosporins, cephamycins and fluoroquinolones, and who had better treatment outcome. A recent study from Taiwan reported that the ESBL positive *E. coli* ST131, which has emerged in bloodstream infections in Taiwan, is not related to more health-care-associated risk factors, and the *E. coli* bacteremia caused by this clone did not exhibited a higher mortality rate [34]. Thus the prevalent clones are not always the most virulent ones or those associated with more severe clinical features or outcome.

Since the prevalent clones have a great potential of transmission among patients, the observation that those clones were significantly associated with MDR, HA infection, as well as nosocomial infections in the crowded ICU, together with the identification of a large variety of drug resistance-associated genes, particularly those ESBL genes, as well as genes associated with resistance to fluoroquinolones and aminoglycosides [35]–[41], in those prevalent clones suggest that although those isolates are associated with less severe clinical features and outcome, they could be a dangerous reservoir for transmission of drug resistance genes, thus warrant a high degree of awareness and monitoring of those drug resistance determinants in clinical isolates. In addition, these isolates from China had different ESBL genotypes, implying multiple acquisition events and the presence of multiple circulating variants of the clone.

In conclusion, the diversity of the genotypes and the complexity of the resistance phenotypes and determinants found, as well as the potential for widespread dissemination of those prevalent isolates detected in our study suggest that certain possibly less virulent (based on the clinical manifestations of the patients) but highly transmissible drug-resistant clones of *K. pneumoniae* isolates are currently prevalent among patients in hospital settings in Beijing, emphasizing the continuous hospital-wide surveillance of phenotypic and genotypic drug resistance data, as well clinical characteristics and treatment outcome for the prevalent *K. pneumoniae* clones is necessary to understand the spread of those successful clones, so as to make better infection control measure against nosocomial infection caused by *K. pneumoniae* and *Enterobacteriaceae*, which are closely related to *K. pneumoniae* and interchange resistance determinants frequently with them. Further in-depth investigation of other important population genetic forces, such as gene flow, natural selection, etc., with more extensive sampling, would validate the interesting observation of an inverse relationship between prevalence and virulence in a statistically robust fashion, as well as to provide more insights into the spatial and temporal population dynamics of drug-resistant *K. pneumoniae* isolates.

## Supporting Information

Table S1 **ST and CC for all** ***K. pneumoniae*** **isolates in this study.**
(DOC)

Table S2 **Variation in loci used in the present** ***K. pneumoniae*** **MLST scheme.**
(DOC)

Table S3 **Drug resistance profiles and epidemiological information of the** ***K. pneumoniae*** **clones belong to phylogenetically distinct cluster of related STs.**
(DOC)

Table S4 **Drug resistance profiles and epidemiological information of the prevalent** ***K. pneumoniae*** **clones.**
(DOC)

Table S5 **Univariate logistic regression analysis for factors associated with prevalent** ***K. pneumoniae*** **clones.**
(DOC)
