# Supplementary material for: Genotypic Analysis of Klebsiella pneumoniae Isolates in a Beijing Hospital Reveals High Genetic Diversity and Clonal Population Structure of Drug-Resistant Isolates
Source: PLoS One. 2013 Feb 21;8(2):e57091. doi: 10.1371/journal.pone.0057091 (PMC3578803; doi:10.1371/journal.pone.0057091)
Supplement: Table S1 — ST and CC for all K. pneumoniae isolates in this study. (DOC) [file pone.0057091.s001.doc]

| **Table S1.** ST and CC for all *K. pneumoniae* isolates in this study. | | | | | | | | | |
| --- | --- | --- | --- | --- | --- | --- | --- | --- | --- |
|  | | | | | | | | | |
| **Isolates** | ***gapA*** | ***infB*** | ***mdh*** | ***pgi*** | ***phoE*** | ***rpoB*** | ***tonB*** | **Sequence types (No. of**  **isolates)** | **Clonal**  **complexes** |
| TZSKP-ATCC 700603 (Reference strain) | 18 | 22 | 26 | 71 | 98 | 52 | 51 | 489 (1) | Singleton |
| TZSKP-110, 211, 218 | 4 | 4 | 1 | 1 | 7 | 4 | 10 | 1 (3) | 1 |
| TZSKP-13, 15, 17, 84, 123, 157, 168, 213, 240 | 3 | 3 | 1 | 1 | 1 | 1 | 4 | 11 (9) | 23 |
| TZSKP-1, 9, 14, 51, 72, 77, 82, 91, 108, 112, 139, 152, 174, 193, 198, 222, 236 | 1 | 1 | 1 | 1 | 1 | 1 | 1 | 15 (17) | 23 |
| TZSKP-61, 67, 129, 184 | 2 | 1 | 1 | 1 | 4 | 4 | 4 | 17 (4) | 23 |
| TZSKP-22, 78, 97, 111, 120, 131, 136, 176, 185, 196, 200, 202 | 2 | 1 | 1 | 1 | 9 | 4 | 12 | 23 (12) | 23 |
| TZSKP-144, 212 | 2 | 1 | 1 | 1 | 10 | 4 | 13 | 25 (2) | 23 |
| TZSKP-220 | 2 | 3 | 2 | 2 | 6 | 4 | 4 | 29 (1) | 29 |
| TZSKP-238 | 2 | 1 | 2 | 1 | 10 | 1 | 19 | 35 (1) | 23 |
| TZSKP-64, 68, 80 | 2 | 1 | 2 | 1 | 7 | 1 | 7 | 36 (3) | 23 |
| TZSKP-6, 28, 76, 117, 214 | 2 | 9 | 2 | 1 | 13 | 1 | 16 | 37 (5) | 37 |
| TZSKP-104, 126, 223 | 2 | 1 | 2 | 1 | 10 | 4 | 13 | 65 (3) | 23 |
| TZSKP-58, 207, 231 | 9 | 4 | 2 | 1 | 1 | 1 | 27 | 86 (3) | 86 |
| TZSKP-210 | 2 | 6 | 1 | 5 | 4 | 1 | 6 | 101 (1) | 101 |
| TZSKP-52, 56, 70, 73, 140, 146, 173, 225 | 3 | 4 | 6 | 1 | 7 | 4 | 38 | 147 (8) | 147 |
| TZSKP-190 | 2 | 3 | 41 | 1 | 17 | 4 | 46 | 189 (1) | Singleton |
| TZSKP-55, 228 | 2 | 3 | 1 | 1 | 9 | 4 | 12 | 218 (2) | 23 |
| TZSKP-221 | 2 | 1 | 1 | 1 | 4 | 27 | 12 | 261 (1) | 23 |
| TZSKP-79 | 2 | 1 | 2 | 1 | 7 | 1 | 81 | 268 (1) | 23 |
| TZSKP-105, 199, 205 | 3 | 4 | 6 | 1 | 7 | 4 | 4 | 273 (3) | 147 |
| TZSKP-32, 237 | 3 | 3 | 1 | 1 | 1 | 1 | 18 | 340 (2) | 23 |
| TZSKP-188 | 2 | 3 | 58 | 37 | 10 | 27 | 9 | 374 (1) | Singleton |
| TZSKP-122, 201, 233 | 43 | 1 | 2 | 1 | 10 | 4 | 13 | 375 (3) | 23 |
| TZSKP-150, 154, 175 | 2 | 1 | 1 | 1 | 1 | 4 | 19 | 380 (3) | Singleton |
| TZSKP-100, 107, 113, 167, 206, 216, 226 | 2 | 1 | 2 | 1 | 9 | 1 | 112 | 412 (7) | 23 |
| TZSKP-227, 229, 239, 241, 242 | 2 | 1 | 1 | 6 | 1 | 4 | 12 | 462 (5) | 23 |
| TZSKP-164, 172 | 38 | 19 | 53 | 58 | 73 | 21 | 130 | 526 (2) | 526-928 |
| TZSKP-7, 18, 21, 38, 40, 69, 93, 94, 103, 118, 130, 145, 149, 166 | 2 | 5 | 1 | 1 | 10 | 1 | 139 | 562 (14) | Singleton |
| TZSKP-48 | 4 | 1 | 2 | 1 | 1 | 7 | 4 | 584 (1) | 584 |
| TZSKP-3 | 2 | 3 | 87 | 1 | 12 | 1 | 26 | 629 (1) | Singleton |
| TZSKP-54 | 2 | 1 | 2 | 1 | 4 | 1 | 25 | 660 (1) | 23 |
| TZSKP-141 | 2 | 1 | 2 | 1 | 3 | 4 | 25 | 685 (1) | 23 |
| TZSKP-47, 65, 66, 135 | 4 | 1 | 1 | 3 | 3 | 5 | 54 | 686 (4) | Singleton |
| TZSKP-62 | 2 | 1 | 2 | 1 | 1 | 4 | 42 | 692 (1) | 23 |
| TZSKP-2, 81, 86, 87, 92, 95, 101, 102, 109, 217 | 71 | 1 | 1 | 2 | 16 | 4 | 164 | 716 (10) | Singleton |
| TZSKP-4 | 2 | 1 | 2 | 1 | 7 | 1 | 12 | 815 (1) | 23 |
| TZSKP-89^a^ | 16 | 18 | 21 | 27 | 39 | 22 | 105 | 856 (1) | 536-856 |
| TZSKP-115^a^ | 2 | 35 | 2 | 35 | 56 | 24 | 19 | 857 (1) | 857 |
| TZSKP-182 | 14 | 1 | 2 | 1 | 7 | 4 | 182 | 873 (1) | Singleton |
| TZSKP-44^a^ | 4 | 1 | 1 | 1 | 7 | 1 | 22 | 874 (1) | 23 |
| TZSKP-74^a^ | 2 | 1 | 2 | 1 | 10 | 4 | 19 | 875 (1) | 23 |
| TZSKP-124^a^ | 6 | 3 | 1 | 1 | 12 | 1 | 110 | 876 (1) | 12 |
| TZSKP-151^a^ | 59 | 24 | 21 | 78 | 54 | 22 | 67 | 877 (1) | Singleton |
| TZSKP-177^a^, 178^a^ | 2 | 5 | 1 | 1 | 144 | 1 | 4 | 878 (2) | Singleton |
| TZSKP-181^a^ | 2 | 1 | 1 | 1 | 145 | 1 | 9 | 879 (1) | 23 |
| TZSKP-183^a^ | 2 | 9 | 2 | 1 | 1 | 1 | 16 | 880 (1) | 37 |
| TZSKP-191^a^ | 2 | 68 | 1 | 1 | 10 | 4 | 13 | 881 (1) | 23 |
| TZSKP-192^a^ | 2 | 1 | 1 | 37 | 1 | 27 | 19 | 882 (1) | Singleton |
| TZSKP-204^a^ | 2 | 5 | 1 | 1 | 9 | 4 | 9 | 883 (1) | Singleton |
| TZSKP-208^a^ | 77 | 9 | 2 | 1 | 13 | 1 | 16 | 884 (1) | 37 |
| TZSKP-127^a^ | 16 | 1 | 21 | 27 | 29 | 17 | 183 | 886 (1) | Singleton |
| TZSKP-128^a^ | 2 | 1 | 1 | 1 | 21 | 4 | 12 | 887 (1) | 23 |
| TZSKP-159^a^ | 2 | 6 | 1 | 5 | 4 | 1 | 4 | 888 (1) | 101 |
| TZSKP-179^a^ | 3 | 1 | 1 | 4 | 3 | 1 | 19 | 889 (1) | 889-7 |
| TZSKP-187^a^ | 2 | 1 | 1 | 8 | 10 | 4 | 61 | 890 (1) | Singleton |
| TZSKP-195^a^ | 3 | 1 | 2 | 1 | 9 | 1 | 184 | 891 (1) | Singleton |
| TZSKP-203^a^ | 2 | 1 | 65 | 2 | 5 | 4 | 36 | 892 (1) | Singleton |
| TZSKP-215^a^ | 25 | 1 | 101 | 1 | 10 | 1 | 100 | 893 (1) | Singleton |
| TZSKP-230^a^ | 18 | 15 | 18 | 61 | 93 | 37 | 99 | 894 (1) | Singleton |
| TZSKP-232^a^ | 3 | 3 | 1 | 1 | 1 | 1 | 42 | 895 (1) | 23 |
| TZSKP-235^a^ | 2 | 9 | 2 | 1 | 13 | 1 | 38 | 896 (1) | 37 |
| ^a^ *K. pneumoniae* isolates which obtained novel STs in this study. | | | | | | | | | |
