# Supplementary material for: Genotypic Analysis of Klebsiella pneumoniae Isolates in a Beijing Hospital Reveals High Genetic Diversity and Clonal Population Structure of Drug-Resistant Isolates
Source: PLoS One. 2013 Feb 21;8(2):e57091. doi: 10.1371/journal.pone.0057091 (PMC3578803; doi:10.1371/journal.pone.0057091)
Supplement: Table S2 — Variation in loci used in the present K. pneumoniae MLST scheme. (DOC) [file pone.0057091.s002.doc]

| **Table S2.** Variation in loci used in the present *K. pneumoniae* MLST scheme. | | | | |
| --- | --- | --- | --- | --- |
|  | | | | |
| **Gene** | **Size (bp)** | **No. of alleles** | **Polymorphic sites (%)** | ***dn*/*ds*** |
| *gapA* | 450 | 15 | 23 (5.1%) | 0.0528 |
| *infB* | 318 | 12 | 23 (7.2%) | 0 |
| *mdh* | 477 | 11 | 48 (10.1%) | 0.0299 |
| *pgi* | 432 | 13 | 39 (9.0%) | 0.0107 |
| *phoE* | 420 | 21 | 35 (8.3%) | 0.0199 |
| *rpoB* | 501 | 10 | 30 (6.0%) | 0.0151 |
| *tonB* | 414 | 34 | 73 (8.2%) | 0.0588 |
|  | | | | |
