# Supplementary material for: Genotypic Analysis of Klebsiella pneumoniae Isolates in a Beijing Hospital Reveals High Genetic Diversity and Clonal Population Structure of Drug-Resistant Isolates
Source: PLoS One. 2013 Feb 21;8(2):e57091. doi: 10.1371/journal.pone.0057091 (PMC3578803; doi:10.1371/journal.pone.0057091)
Supplement: Table S3 — Drug resistance profiles and epidemiological information of the K. pneumoniae clones belong to phylogenetically distinct cluster of related STs. (DOC) [file pone.0057091.s003.doc]

| **Table S3.** Drug resistance profiles and epidemiological information of the *K. pneumoniae* clones belong to phylogenetically distinct cluster of related STs ^a^. | | | | | | | |
| --- | --- | --- | --- | --- | --- | --- | --- |
|  | | | | | | | |
| **Isolates** | **Phenotypic drug resistance ^b^** | **Drug resistance genes** | **ESBL** | **Infection acquired model** | **Diagnosis** | **Date of hospitalization** | **Hospital location** |
| TZSKP-164 | AMP,CRO,CAZ,FEP,ATM,CIP,LVX,GM,TOB,SXT,FD | *bla*_CTX-M-1_, *bla*_CTX-M-3_, *bla*_SHV-11_, *bla*_DHA-1_, *aac(6’)-Ib-cr* | + | HA | Pulmonary infection | 2011.7.2-2011.8.28 | Department of respiration |
| TZSKP-172 | AMP,CFZ,CRO,CAZ,FEP,ATM,CIP,LVX,GM,TOB,SXT,FD | *bla*_CTX-M-1_, *bla*_CTX-M-3_, *bla*_CTX-M-10_, *bla*_TEM-1_,*qnrB*,*qnrD*,*qnrS*,*aac(6’)-Ib-cr*,*aacA4*,*aacC2*,*aadA1* | + | HA | Chronic obstructive pulmonary disease | 2011.7.20-2011.9.8 | Department of respiration |
| TZSKP-151^c^ | AMP | None | - | CA | Pneumonia | 2011.7.3-2011.7.19 | Department of Pediatric Hematology |
| TZSKP-127^c^ | AMP,FD | None | - | HA | [Postpartum hemorrhage](app:ds:postpartum hemorrhage) | 2011.6.3-2011.6.7 | Department of gynaecology and obstetrics |
| TZSKP-89^c^ | AMP | None | - | HA | hepatic encephalopathy | 2011.4.17-2011.6.4 | Department of gastroenterology |
| TZSKP-230^c^ | AMP,CTT,FD | None | + | CA | No data | No data | No data |
| ^a^ *K. pneumoniae* clones belonging to phylogenetically distinct cluster of related STs include: ST526 (TZSKP-164, TZSKP-172), ST877 (TZSKP-151), ST886 (TZSKP-127), ST856 (TZSKP-89), and ST894 (TZSKP-230).  ^b^ Abbreviation of antibiotics: AMP, Ampicillin; TZP, Piperacillin/Tazobactam; SAM, Ampicillin/Sulbactam; CFZ, Cefazolin; CRO, Ceftriaxone; CAZ, Ceftazidime; FEP, Cefepime; CTT, Cefotetan; ETP, Ertapenem; IMP, Imipenem; ATM, Aztreonam; CIP, Ciprofloxacin; LVX, Levofloxacin; GM, Gentamycin; TOB, Tobramycin; AMK, Amikacin; SXT, Trimethoprim-Sulfamethoxazole; FD, Nitrofurantoin.  ^c^ *K. pneumoniae* isolates which obtained novel STs in this study. | | | | | | | |
