# Supplementary material for: Genotypic Analysis of Klebsiella pneumoniae Isolates in a Beijing Hospital Reveals High Genetic Diversity and Clonal Population Structure of Drug-Resistant Isolates
Source: PLoS One. 2013 Feb 21;8(2):e57091. doi: 10.1371/journal.pone.0057091 (PMC3578803; doi:10.1371/journal.pone.0057091)
Supplement: Table S4 — Drug resistance profiles and epidemiological information of the prevalent K. pneumoniae clones. (DOC) [file pone.0057091.s004.doc]

| **Table S4.** Drug resistance profiles and epidemiological information of the prevalent *K. pneumoniae* clones. | | | | | | | | |
| --- | --- | --- | --- | --- | --- | --- | --- | --- |
|  | | | | | | | | |
| **Isolates** | **Phenotypic drug resistance^a^** | **Drug resistance genes** | **ESBL** | | **Infection acquired model** | **Diagnosis** | **Date of hospitalization** | **Hospital location** |
| ST15 (17) | | | | | | | | |
| TZSKP-1 | AMP,TZP,SAM,CFZ,CRO,CAZ,FEP,CTT,ETP,IMP,ATM,CIP,LVX,GM,TOB,AMK,SXT,FD | *bla*_CTX-M-3_,*bla*_CTX-M-10_,*bla*_SHV-11_,*dhfr*,*qnrB*,*aac(6’)-Ib-cr*,*aacA4*,*aacC2* | + | | CA | Septic shock, pneumonia | 2011.8.7-2011.9.1 | The first Ward, Department of respiration |
| TZSKP-9 | AMP,SAM,CFZ,CRO,CAZ,FEP,ATM,CIP,LVX,SXT,FD | *bla*_CTX-M-14_,*dhfr* | + | | CA | Pneumonia | 2010.11.18-2010.12.14 | The first Ward, Department of geratology |
| TZSKP-14 | AMP,SAM,CFZ,CRO,CAZ,FEP,ATM,CIP,LVX,SXT,FD | *bla*_SHV-1_,*dhfr* | + | | CA | Pulmonary infection | 2010.11.8-2010.11.28 | The first Ward, Department of geratology |
| TZSKP-51 | AMP,TZP,SAM,CFZ,CRO,CAZ,FEP,CTT,ATM,CIP,LVX,GM,TOB,AMK,SXT,FD | *bla*_CTX-M-15_,*bla*_SHV-85_,*dhfr*,*qnrS*,*aac(6’)-Ib-cr*,*aacC2*,*aadA1*,*aadB* | + | | HA | Renal dysfunction | 2011.2.9-2011.3.26 | The first Ward, Department of respiration |
| TZSKP-72 | AMP,FEP,CTT,ETP,IMP,ATM,CIP,LVX,GM,TOB,SXT,FD | *bla*_CTX-M-15_,*aacA4* | + | | HA | Heart failure | 2011.3.26-2011.4.1 | Cardiac care unit |
| TZSKP-77 | AMP,TZP,SAM,CFZ,CRO,CAZ,FEP,CTT,ATM,CIP,LVX,GM,TOB,AMK,SXT,FD | *qnrB*,*aacA4* | + | | HA | Bronchial asthma | 2011.4.21-2011.5.12 | The first Ward, Department of respiration |
| TZSKP-82 | AMP,TZP,SAM,CFZ,CRO,CAZ,FEP,CTT,ATM,CIP,LVX,GM,TOB,AMK,SXT,FD | *bla*_CTX-M-3_,*bla*_CTX-M-9_,*bla*_CTX-M-10_,*bla*_OXA-48_,*aac(6’)-Ib-cr*,*aacA4*,*aacC2* | + | | CA | Chronic obstructive pulmonary disease | 2011.4.8-2011.4.15 | Neuro-intensive care unit |
| TZSKP-91 | AMP,SAM,CFZ,CRO,CAZ,FEP,CTT,ATM,CIP,LVX,TOB,AMK,SXT,FD | *bla*_CTX-M-3_,*bla*_CTX-M-10_,*bla*_CTX-M-55_,*bla*_TEM-186_,*dhfr*,*qnrB*,*aac(6’)-Ib-cr*,*aacA4*,*aacC2*,*aadA1*,*aadB* | + | | CA | Decubital ulcer | 2011.4.6-2011.5.4 | Emergency internal medicine |
| TZSKP-108 | AMP,TZP,SAM,CFZ,CRO,CAZ,FEP,CTT,ATM,CIP,LVX,GM,TOB,AMK,SXT,FD | *bla*_CTX-M-3_,*bla*_CTX-M-8_,*bla*_CTX-M-10_,*bla*_CTX-M-25_,*dhfr*,*qnrB*,*qnrD*,*aac(6’)-Ib-cr*,*aacA4*,*aadA1* | + | | HA | Coronary heart disease | 2011.5.7-2011.5.20 | Cardiac care unit |
| TZSKP-112 | AMP,TZP,SAM,CFZ,CRO,CAZ,FEP,CTT,ATM,CIP,LVX,GM,TOB,AMK,SXT,FD | *bla*_CTX-M-1_,*bla*_CTX-M-3_,*bla*_CTX-M-10_,*bla*_TEM-1_,*bla*_SHV-1_,*dhfr*,*qnrD*,*aac(6’)-Ib-cr*,*aacA4*,*aacC2*,*aadA1* | + | | CA | Heart failure | 2011.5.21-2011.6.20 | Cardiac care unit |
| TZSKP-139 | AMP,SAM,CFZ,CRO,CAZ,FEP,CTT,ATM,CIP,LVX,GM,TOB,AMK,SXT,FD | *bla*_CTX-M-1_,*qnrB*,*aacA4* | + | | HA | Nasopharyngeal carcinoma | 2011.7.29-2011.8.26 | Department of Hematology and Oncology |
| TZSKP-152 | AMP,SAM,CFZ,CRO,CAZ,FEP,CTT,ATM,CIP,LVX,GM,TOB,AMK,SXT,FD | *bla*_CTX-M-3_,*bla*_CTX-M-8_,*bla*_CTX-M-10_,*bla*_TEM-1_*,dhfr*,*qnrB*,*qnrD*,*aac(6’)-Ib-cr*,*aacA4*,*aacC1*,*aacC2*,*aadA1* | + | | HA | Septic shock | 2011.4.9-2011.7.19 | Intensive care unit |
| TZSKP-174 | AMP,TZP,SAM,CRO,CAZ,FEP,CTT,ATM,CIP,LVX,GM,TOB,AMK,SXT,FD | *bla*_CTX-M-1_,*bla*_CTX-M-3_,*bla*_CTX-M-10_,*bla*_SHV-11_,*ac(6’)-Ib-cr*,*aacA4*,*aacC2* | - | | CA | Chronic obstructive pulmonary disease, pulmonary infection | 2011.6.29-2011.7.22 | The first Ward, Department of respiration |
| TZSKP-193 | AMP,TZP,SAM,CFZ,CRO,CAZ,FEP,CTT,ATM,CIP,LVX,GM,TOB,AMK,SXT,FD | *bla*_CTX-M-1_,*bla*_CTX-M-3_,*bla*_CTX-M-8_,*bla*_CTX-M-10_,*bla*_CTX-M-14_,*bla*_SHV-1_,*bla*_TEM-1_*,dhfr*,*qnrB*,*qnrD*,*aac(6’)-Ib-cr*,*aacA4*,*aacC2*,*aadA1* | + | | HA | Omphalitis of newborn | 2011.8.28-2011.9.1 | Department of Pediatric Hematology |
| TZSKP-198 | AMP,SAM,CFZ,CRO,CAZ,FEP,CTT,ATM,CIP,LVX,GM,TOB,AMK,SXT,FD | *bla*_CTX-M-10_,*bla*_SHV-11_,*bla*_TEM-1_,*dhfr*,*qnrB*,*qnrD*,*aac(6’)-Ib-cr*,*qepA*,*aacA4*,*aacC2*,*aadA1* | + | | HA | Hypertension | 2011.7.8-2011.9.12 | Cardiac care unit |
| TZSKP-222 | AMP,SAM,CFZ,CRO,CAZ,FEP,CTT,ATM,CIP,LVX,GM,TOB,AMK,SXT,FD | *bla*_CTX-M-3_,*bla*_CTX-M-8_,*bla*_CTX-M-10_*,bla*_SHV-1_,*dhfr*,*qnrB*,*qnrD*,*aac(6’)-Ib-cr*,*aacA4*,*aadA1*,*aphA6* | + | | HA | Pericardial effusion | 2011.7.21-2011.10.13 | Cardiac care unit |
| TZSKP-236 | AMP,TZP,SAM,CFZ,CRO,CAZ,FEP,CTT,ATM,CIP,LVX,GM,TOB,SXT,FD | *bla*_CTX-M-3_,*bla*_CTX-M-10_,*bla*_SHV-11_,*bla*_TEM_,*dhfr*,*qnrB*,*aac(6’)-Ib-cr*,*aacA4*,*aacC1*,*aacC2*,*aadA1,armA* | + | | HA | Cerebral hemorrhage | No data | No data |
| ST562 (14) | | | | | | | | |
| TZSKP-7 | AMP,TZP,CFZ,CRO,CAZ,FEP,CTT,ATM,GM,TOB,AMK,SXT,FD | *bla*_CTX-M-14_,*bla*_SHV-11_,*bla*_TEM-1_,*qnrS*,*aacC2* | | - | HA | Respiratory failure, Cerebral infarction | 2010.11.15-2010.12.2 | Neuro-intensive care unit |
| TZSKP-18 | AMP,SAM,CFZ,CRO,CAZ,FEP,ATM,GM,TOB,SXT,FD | *bla*_CTX-M-10_,*bla*_SHV-85_,*qnrS*,*aacC2* | | + | HA | Subarachnoid hemorrhage | 2010.11.24-2010.12.2 | Neuro-intensive care unit |
| TZSKP-21 | AMP,SAM,CFZ,CRO,CAZ,FEP,ATM,GM,TOB,SXT,FD | *bla_SHV-85_*,*dhfr*,*qnrS*,*aacC2* | | + | HA | Left femoral neck fracture | 2010.11.1-2010.12.20 | Intensive care unit |
| TZSKP-38 | AMP,SAM,CFZ,CRO,CAZ,FEP,CTT,ATM,GM,TOB,SXT,FD | *bla*_CTX-M-1_,*bla*_CTX-M-10_,*bla*_CTX-M-15_,*bla*_TEM_,*qnrS*,*aacC2* | | + | HA | Subarachnoid hemorrhage | 2010.12.7-2011.1.27 | Neuro-intensive care unit |
| TZSKP-40 | AMP,SAM,CFZ,CRO,CAZ,FEP,ATM,GM,TOB,SXT,FD | *bla*_CTX-M-14_,*bla*_CTX-M-10_,*bla*_SHV-11_,*bla*_TEM-1_,*qnrS*,*aacC2* | | + | HA | Cerebral infarction | 2011.1.12-2011.2.11 | The second Ward, Department of respiration |
| TZSKP-69 | AMP,SAM,CFZ,CRO,CAZ,FEP,ATM,GM,TOB,SXT,FD | *bla*_SHV-11_,*qnrS*,*aacC2,armA* | | + | HA | Parkinsonism | 2011.1.15-2011.4.19 | The second Ward, Department of respiration |
| TZSKP-93 | AMP,SAM,CFZ,CRO,CAZ,FEP,ATM,CIP,LVX,GM,TOB,SXT,FD | *bla*_CTX-M-1_,*bla*_CTX-M-14_,*bla*_TEM-1_,*dhfr*,*aacA4* | | + | HA | Cerebral infarction | 2011.4.28-2011.5.24 | Neuro-intensive care unit |
| TZSKP-94 | AMP,SAM,CFZ,CRO,CAZ,FEP,ATM,GM,TOB,SXT,FD | *qnrB*,*aac(6’)-Ib-cr*,*aacA4*,*aacC2* | | + | HA | Cerebral abscess | 2011.4.26-2011.5.31 | Neuro-intensive care unit |
| TZSKP-103 | AMP,SAM,CFZ,CRO,CAZ,FEP,ATM,GM,TOB,SXT,FD | *bla*_CTX-M-14_,*bla*_CTX-M-10_*,bla*_CTX-M-15_,*bla*_SHV-1_,*dhfr*,*qnrD*,*qnrS*,*aadA1* | | + | HA | Subarachnoid hemorrhage | 2011.5.7-2011.6.6 | Neuro-intensive care unit |
| TZSKP-118 | AMP,SAM,CFZ,CRO,CAZ,FEP,ATM,SXT,FD | *bla*_CTX-M-10_,*bla*_CTX-M-25_,*qnrS,aacC2* | | + | HA | Intracranial infection | 2011.5.17-2011.7.6 | Neuro-intensive care unit |
| TZSKP-130 | AMP,TZP,SAM,CFZ,CRO,CAZ,FEP,ATM,GM,TOB,SXT,FD | *bla*_CTX-M-1_,*bla*_CTX-M-3_,*bla*_CTX-M-14_,*bla*_CTX-M-10_,*dhfr*,*qnrC*,*qnrS*,*qepA*,*aacC2*,*aadA1* | | + | HA | Cerebral infarction | 2011.6.5-2011.7.12 | Neuro-intensive care unit |
| TZSKP-145 | AMP,SAM,CFZ,CRO,CAZ,FEP,ATM,GM,TOB,SXT,FD | *bla*_CTX-M-14_,*bla*_CTX-M-10_,*bla*_SHV-1_,*bla*_TEM-1_,*dhfr*,*qnrB*,*qnrD*,*qnrS*,*aac(6’)-Ib-cr*,*aacC2*,*aadA1* | | + | CA | Pneumonia | 2011.5.31-2011.7.3 | The second Ward, Department of respiration |
| TZSKP-149 | AMP,SAM,CFZ,CRO,CAZ,FEP,ATM,GM,TOB,SXT,FD | *qnrS* | | + | HA | Hypoxic ischemic encephalopathy | 2011.4.25-2011.6.24 | Neuro-intensive care unit |
| TZSKP-166 | AMP,SAM,CFZ,CRO,CAZ,FEP,CTT,ATM,CIP,LVX,GM,TOB,AMK,SXT,FD | *bla*_CTX-M-1_,*bla*_SHV-85_ | | + | HA | Nasopharyngeal carcinoma | 2011.8.28-2011.9.10 | Department of Hematology and Oncology |
| ST23 (12) | | | | | | | | |
| TZSKP-22 | AMP,TZP,SAM,CTT,CIP,LVX,TOB,AMK,SXT,FD | *bla*_SHV-11_ | | - | CA | Pulmonary infection | 2011.4.2-2011.4.9 | The second Ward, Department of respiration |
| TZSKP-78 | AMP,TZP,SAM,CTT,CIP,LVX,TOB,AMK,SXT,FD | *bla*_SHV-85_,*qnrS,aac(6’)-Ib-cr,aacA4* | | - | CA | Chronic obstructive pulmonary disease | 2011.4.2-2011.4.9 | The second Ward, Department of respiration |
| TZSKP-97 | AMP,CTT,FD | *qnrD* | | - | HA | Liver abscess | 2011.4.29-2011.6.1 | The first Ward, Department of geratology |
| TZSKP-111 | AMP,CTT | *qnrD,aacC2* | | - | HA | Cerebral infarction | 2011.11.11-2011.11.18 | Intensive care unit |
| TZSKP-120 | AMP,CTT,FD | *qnrD* | | - | HA | Renal dysfunction | 2011.11.28-2011.12.8 | Intensive care unit |
| TZSKP-131 | AMP,CTT,FD | None | | - | CA | Lung cancer | 2011.6.6-2011.7.8 | The first Ward, Department of geratology |
| TZSKP-136 | AMP,CTT,FD | None | | - | HA | Cerebral infarction | 2011.11.11-2011.11.18 | Intensive care unit |
| TZSKP-176 | AMP,CTT,FD | *qnrD*,*aadA1* | | - | HA | Neonatal hyperbilirubinemia | 2011.8.7-2011.8.16 | Department of Pediatric Hematology |
| TZSKP-185 | AMP,CTT,FD | None | | - | CA | Tuberculous pleurisy | 2011.8.17-2011.9.22 | The first Ward, Department of respiration |
| TZSKP-196 | AMP,CTT,FD | None | | - | HA | Cerebral infarction | 2011.9.8-2011.10.23 | Intensive care unit |
| TZSKP-200 | AMP,CTT,FD | None | | - | CA | Pneumonia | 2011.9.5-2011.9.14 | The second Ward, Department of respiration |
| TZSKP-202 | AMP,CTT,FD | None | | - | HA | Congestive heart failure | 2011.9.18-2011.11.3 | Intensive care unit |
| ST716 (10) | | | | | | | | |
| TZSKP-2 | AMP,TZP,SAM,CFZ,CRO,CAZ,FEP,CTT,ATM,CIP,LVX,GM,TOB,SXT,FD | *bla*_CTX-M-15_,*bla*_SHV-1_*,**dhfr*,*qnrS*,*aacC2*,*aadA1* | | + | HA | Cerebral infarction | 2010.11.4-2010.11.13 | Neuro-intensive care unit |
| TZSKP-87 | AMP,TZP,SAM,CFZ,CRO,CAZ,FEP,ATM,CIP,LVX,GM,TOB,SXT,FD | *bla*_CTX-M-8_,*bla*_CTX-M-15_,*bla*_SHV-1_,*dhfr*,*qnrS*,*aacC2*, *aadA1* | | + | HA | Bleeding duodenal ulcer | 2011.4.7-2011.5.11 | The second Ward, Department of General Surgery |
| TZSKP-92 | AMP,CTT,CIP,LVX,GM,TOB,SXT,FD | *bla*_SHV-85_,*qnrS,aacC2* | | - | HA | Obstructive jaundice | 2011.4.1-2011.6.14 | The second Ward, Department of General Surgery |
| TZSKP-95 | AMP,TZP,SAM,CFZ,CRO,CAZ,FEP,CTT,ATM,CIP,LVX,GM,TOB,SXT,FD | *bla*_CTX-M-8_,*qnrS,aacC2* | | + | HA | Hemorrhagic shock | 2011.4.24-2011.6.11 | Intensive care unit |
| TZSKP-101 | AMP,CTT,CIP,LVX,GM,TOB,SXT,FD | *qnrS,aacC2* | | - | HA | Thyroid neoplasm | 2011.4.14-2011.6.14 | The second Ward, Department of General Surgery |
| TZSKP-102 | AMP,CTT,CIP,LVX,GM,TOB,SXT,FD | *bla*_CTX-M-8_,*bla*_CTX-M-15_,*qnrS* | | - | HA | Intestinal obstruction | 2011.4.18-2011.7.14 | The second Ward, Department of General Surgery |
| TZSKP-109 | AMP,CTT,CIP,LVX,GM,TOB,SXT,FD | *qnrS,aacC2* | | - | HA | Obstructive jaundice | 2011.4.28-2011.7.2 | The second Ward, Department of General Surgery |
| TZSKP-217 | AMP,SAM,CFZ,CRO,CAZ,FEP,CTT,ATM,CIP,LVX,GM,TOB,AMK,SXT,FD | *bla*_CTX-M-8_,*bla*_CTX-M-9_,*bla*_SHV-11_,*dhfr*,*qnrD*,*qnrS*,*aacC2*,*aadA1* | | + | HA | Prostatic hyperplasia | 2011.8.4-2011.9.28 | Department of Urology |
| TZSKP-81 | AMP,SAM,CFZ,CRO,CAZ,FEP,ATMGM,TOB,SXT,FD | *bla*_SHV-85_,*qnrS*,*qnrS*, *aadA1,armA* | | + | HA | Hypertension | 2011.3.7-2011.8.24 | Department of Neurological Surgery |
| TZSKP-86 | AMP,TZP,SAM,CFZ,CRO,CAZ,FEP,CTT,,ATM,CIP,LVX,GM,TOB,SXT,FD | *bla*_SHV-1_,*qnrS*,*aacC2*,*aadA1* | | + | HA | Bleeding duodenal ulcer | 2011.4.7-2011.5.11 | The second Ward, Department of General Surgery |
| ST11 (9) | | | | | | | | |
| TZSKP-13 | AMP,SAM,CFZ,CRO,CAZ,FEP,ATM,CIP,LVX,TOB,AMK,SXT,FD | *bla*_CTX-M-14_,*dhfr*,*qnrB,aacA4* | | + | CA | Chronic obstructive pulmonary disease | 2011.5.12-2011.6.24 | The first Ward, Department of respiration |
| TZSKP-15 | AMP,SAM,CFZ,CRO,CAZ,FEP,CTT,ETP,IMP,ATM,CIP,LVX,GM,TOB,AMK,SXT,FD | *bla*_CTX-M-1_,*bla*_CTX-M-14_,*bla*_CTX-M-10_,*bla*_SHV-11_,*bla*_TEM_,*dhfr*,*qnrA*,*qnrB,aac(6’)-Ib-cr*,*aacA4*,*aadA1* | | + | CA | Pneumonia | 2011.5.22-2011.7.4 | The first Ward, Department of respiration |
| TZSKP-17 | AMP,TZP,SAM,CFZ,CRO,CAZ,FEP,CTT,ATM,CIP,LVX,GM,TOB,AMK,SXT,FD | *bla*_CTX-M-2_,*bla*_CTX-M-14_,*bla*_CTX-M-10_,*bla*_SHV-11_,*bla*_TEM-1_,*bla*_CMY2_,*bla*_DHA1_,*dhfr*,*qnrS*,*aac(6’)-Ib-cr*,*aacA4*,*aacC2*,*aadA1* | | + | HA | Urinary tract infection | 2011.8.8-2011.8.13 | Department of Urology |
| TZSKP-84 | AMP,CFZ,CRO,CAZ,FEP,CTT,ATM,CIP,LVX,TOB,AMK,SXT,FD | *bla*_SHV-1_,*qnrB*,*aac(6’)-Ib-cr*,*aadA1* | | - | CA | No data | No data | No data |
| TZSKP-123 | AMP,TZP,SAM,CTT,CIP,LVX,FD | None | | - | HA | Esophagitis | 2011.6.10-2011.7.23 | Department of gastroenterology |
| TZSKP-157 | AMP,SAM,CFZ,CRO,CAZ,FEP,ATM,CIP,LVX,GM,TOB,AMK,SXT,FD | *bla*_CTX-M-3_,*bla*_CTX-M-9_,*bla_CTX-M-10_,bla*_SHV-1_,*bla*_TEM-1_,*bla*_DHA-1_,*dhfr*,*qnrB*,*qnrD*,*aac(6’)-Ib-cr*,*aacA4*,*aacC2*,*aadA1**,armA* | | + | HA | Empyema | 2011.6.10-2011.7.29 | Department of Cardiothoracic Surgery |
| TZSKP-168 | AMP,SAM,CFZ,CRO,CAZ,FEP,ATM,CIP,LVX,GM,TOB,AMK,SXT,FD | *bla*_DHA-1_,*qnrB*,*qnrD*,*aacA4*,*aadA1,armA* | | + | HA | Pericardial effusion | 2011.6.30-2011.8.9 | Department of Cardiothoracic Surgery |
| TZSKP-213 | AMP,SAM,CFZ,CRO,CAZ,FEP,CTT,ATM,CIP,LVX,TOB,AMK,SXT,FD | *bla*_CTX-M-14_,*bla*_CTX-M-10_,*bla*_SHV-11_,*dhfr*,*qnrB*,*aac(6’)-Ib-cr*,*aacC2*,*aadA1* | | + | HA | Bladder cancer | 2011.9.14-2011.10.13 | Department of Urology |
| TZSKP-240 | AMP,TZP,SAM,CFZ,CRO,CAZ,FEP,CTT,ATM,CIP,LVX,TOB,AMK,SXT,FD | *bla*_CTX-M-1_,*bla*_CTX-M-3_,*bla*_CTX-M-8_,*bla*_CTX-M-10_,*bla*_SHV-11_,*bla*_TEM-1_,*dhfr*,*aac(6’)-Ib-cr*,*aacA4*,*aacC2*,*aadA1* | | - | CA | Pneumonia | 2011.9.9-2011.11.3 | The second Ward, Department of respiration |
| ST147 (8) | | | | | | | | |
| TZSKP-52 | AMP,TZP,SAM,CFZ,CTT,CIP,LVX,TOB,AMK,SXT,FD | *bla*_CTX-M-9_, *bla*_CTX-M-15_ | | - | CA | Cerebral infarction | 2011.5.26-2011.6.12 | The first Ward, Department of geratology |
| TZSKP-56 | AMP,SAM,CFZ,CRO,CAZ,FEP,ATM,CIP,LVX,GM,TOB,SXT,FD | *bla*_CTX-M-15_ | | + | CA | Pulmonary infection | 2011.2.25-2011.3.11 | Department of traditional Chinese medicine |
| TZSKP-70 | AMP,TZP,SAM,CFZ,CRO,CAZ,FEP,ATM,CIP,LVX,GM,TOB,AMK,SXT,FD | *bla*_CTX-M-15_ | | + | HA | Cerebral infarction | 2011.3.31-2011.7.21 | The first Ward, Department of geratology |
| TZSKP-73 | AMP,CTT,FD | None | | - | CA | Pneumonia | 2011.3.25-2011.7.21 | The first Ward, Department of geratology |
| TZSKP-140 | AMP,TZP,SAM,CFZ,CRO,CAZ,FEP,ATM,CIP,LVX,TOB,AMK,SXT,FD | *bla*_CTX-M-1_,*bla*_CTX-M-8_,*bla*_SHV-1_,*dhfr*,*qnrB*,*qnrD*,*aac(6’)-Ib-cr*,*aacA4*,*aacC2*,*aadA1* | | + | HA | Cerebral infarction | 2011.3.11-2011.6.29 | The first Ward, Department of geratology |
| TZSKP-146 | AMP,TZP,SAM,CFZ,CRO,CAZ,FEP,ATM,CIP,LVX,GM,TOB,AMK,SXT,FD | *bla*_CTX-M-1_*,bla*_CTX-M-3_,*bla*_CTX-M-9_*,bla*_CTX-M-10_,*bla*_SHV-11_,*bla*_TEM_,*dhfr,qnrD,aac(6’)-Ib-cr*,*aacA4*,*aacC2,aadA1* | | + | CA | Chronic obstructive pulmonary disease | 2011.6.8-2011.6.29 | The first Ward, Department of geratology |
| TZSKP-173 | AMP,TZP,SAM,CFZ,CRO,CAZ,FEP,ATM,CIP,LVX,GM,TOB,AMK,SXT,FD | *bla*_CTX-M-1_,*bla*_CTX-M-3_,*bla*_CTX-M-8_,*bla*_CTX-M-10_,*bla*_SHV-11_,*bla*_TEM-1_,*dhfr*,*qnrD*,*aac(6’)-Ib-cr*,*aacA4* | | + | HA | Pulmonary infection | 2011.6.28-2011.7.27 | The first Ward, Department of geratology |
| TZSKP-225 | AMP,TZP,SAM,CFZ,CRO,CAZ,FEP,CTT,ATM,CIP,LVX,TOB,AMK,SXT,FD | *bla*_CTX-M-_*_1_*,*bla*_CTX-M-3_,*bla*_CTX-M-9_,*bla*_CTX-M-10_,*bla*_TEM-1_,*dhfr*,*qnrD*,*aac(6’)-Ib-cr*,*aacA4*,*aacC2*,*aadA1* | | - | HA | Pneumonia | 2011.6.22-2011.8.2 | The first Ward, Department of respiration |
| ^a^ Abbreviation of antibiotics: AMP, Ampicillin; TZP, Piperacillin/Tazobactam; SAM, Ampicillin/Sulbactam; CFZ, Cefazolin; CRO, Ceftriaxone; CAZ, Ceftazidime; FEP, Cefepime; CTT, Cefotetan; ETP, Ertapenem; IMP, Imipenem; ATM, Aztreonam; CIP, Ciprofloxacin; LVX, Levofloxacin; GM, Gentamycin; TOB, Tobramycin; AMK, Amikacin; SXT, Trimethoprim-Sulfamethoxazole; FD, Nitrofurantoin. | | | | | | | | |
