# Supplementary material for: Genotypic Analysis of Klebsiella pneumoniae Isolates in a Beijing Hospital Reveals High Genetic Diversity and Clonal Population Structure of Drug-Resistant Isolates
Source: PLoS One. 2013 Feb 21;8(2):e57091. doi: 10.1371/journal.pone.0057091 (PMC3578803; doi:10.1371/journal.pone.0057091)
Supplement: Table S5 — Univariate logistic regression analysis for factors associated with prevalent K. pneumoniae clones. (DOC) [file pone.0057091.s005.doc]

| **Table S5.** Univariate logistic regression analysis for factors associated with prevalent *K. pneumoniae* clones^a^. | | | | |
| --- | --- | --- | --- | --- |
|  | | | | |
| **Variables** | **Prevalent clones, n/N (%)^a^** | **Odds Ratio** | **95% CI** | ***P* value** |
| Gender |  |  |  |  |
| Male | 50/120 (41.7) | 1 |  |  |
| Female | 20/43 (46.5) | 1.217 | 0.604-2.453 | 0.582 |
| Age group, years |  |  |  |  |
| <15 | 2/6 (33.3) | 0.654 | 0.116-3.678 | 0.630 |
| 15-60 | 14/31 (45.2) | 1.118 | 0.509-2.455 | 0.782 |
| >60 | 54/126 (42.9) | 0.984 | 0.470-2.063 | 0.967 |
| Residence situation |  |  |  |  |
| Beijing resident | 19/54 (35.2) | 1 |  |  |
| Non-Beijing resident | 51/109 (46.8) | 0.617 | 0.315-1.211 | 0.160 |
| Hospital location |  |  |  |  |
| Emergency room | 2/8 (25.0) | 0.426 | 0.083-2.180 | 0.306 |
| Intensive care unit | 25/49 (51.0) | 1.597 | 0.814-3.135 | 0.174 |
| Medical ward | 34/75 (45.3) | 1.198 | 0.643-2.232 | 0.570 |
| Surgical ward | 9/31 (29.0) | 0.476 | 0.204-1.111 | 0.086 |
| Sources of specimens |  |  |  |  |
| Sputum | 52/121 (43.0) | 1.005 | 0.494-2.042 | 0.989 |
| Urine | 6/12 (50.0) | 1.359 | 0.419-4.409 | 0.609 |
| Throat or nose swabs | 2/13 (15.4) | 0.219 | 0.047-1.023 | 0.054 |
| Blood | 5/8 (62.5) | 2.308 | 0.532-10.001 | 0.264 |
| Others | 5/9 (55.6) | 1.712 | 0.442-6.623 | 0.436 |
| Underlying diseases |  |  |  |  |
| Pneumonia | 7/14 (50.0) | 1.333 | 0.444-4.005 | 0.608 |
| Diabetes mellitus | 19/40 (47.5) | 1.244 | 0.603-2.565 | 0.554 |
| Chronic bronchitis | 6/11 (54.6) | 1.613 | 0.470-5.531 | 0.447 |
| Chronic obstructive pulmonary disease | 6/14 (42.9) | 0.971 | 0.320-2.947 | 0.959 |
| A[bnormal](http://www.iciba.com/abnormal/) liver function | 19/36 (52.8) | 1.630 | 0.770-3.452 | 0.202 |
| Renal dysfunction | 18/35 (51.4) | 1.513 | 0.710-3.224 | 0.284 |
| Hypertension | 35/84 (41.7) | 0.848 | 0.447-1.608 | 0.614 |
| Heart disease | 22/55 (40.0) | 0.800 | 0.410-1.562 | 0.513 |
| Cerebral infarction | 21/41 (51.2) | 1.529 | 0.746-3.136 | 0.246 |
| Pulmonary infection | 37/81 (45.7) | 1.205 | 0.636-2.284 | 0.567 |
| Urinary tract infection | 4/11 (36.4) | 0.735 | 0.206-2.621 | 0.635 |
| Infection acquired model |  |  |  |  |
| Community acquired | 20/37 (54.1) | 1.788 | 0.854-3.742 | 0.123 |
| Hospital acquired | 50/126 (39.7) | 0.559 | 0.267-1.170 | 0.123 |
| ESBL |  |  |  |  |
| Negative | 24/83 (28.9) | 1 |  |  |
| Positive | 46/80 (57.5) | 0.301 | 0.157-0.576 | <0.001 |
| Drug resistance profiles |  |  |  |  |
| Penicillins | 70/162 (43.2) | 0.000 | 0.000- | 1.000 |
| 1st and 2nd generation cephalosporins | 51/86 (59.3) | 0.225 | 0.115-0.441 | <0.001 |
| 3rd and 4th generation cephalosporins | 51/86 (59.3) | 0.225 | 0.115-0.441 | <0.001 |
| Cephamycins | 47/108 (43.5) | 0.933 | 0.484-1.800 | 0.836 |
| Carbapenems | 7/12 (58.3) | 0.511 | 0.155-1.685 | 0.270 |
| Monobactams | 50/83 (60.2) | 0.220 | 0.113-0.430 | <0.001 |
| Fluoroquinolones | 47/72 (65.3) | 0.180 | 0.091-0.354 | <0.001 |
| Aminoglycosides | 56/90 (62.2) | 0.144 | 0.070-0.297 | <0.001 |
| Folate pathway inhibitors | 57/94 (60.6) | 0.151 | 0.072-0.313 | <0.001 |
| Nitrofurantoin | 69/145 (47.6) | 0.065 | 0.008-0.500 | 0.009 |
| Drug resistance types |  |  |  |  |
| MDR | 47/104 (45.2) | 1.291 | 0.674-2.473 | 0.442 |
| XDR | 17/34 (50.0) | 1.434 | 0.672-3.061 | 0.352 |
| Treatment outcome |  |  |  |  |
| Treatment success | 61/138 (44.2) | 1.408 | 0.582-3.406 | 0.447 |
| Death | 9/25 (36.0) | 0.710 | 0.294-1.717 | 0.447 |
| **^a^** Prevalent clones include a total of 70 isolates with the following STs: ST15 (17), ST562 (14), ST23 (12), ST716 (10), ST11 (9), ST147 (8). | | | | |
